# Supplementary material for: A cross-sectional questionnaire study of the rules governing pupils’ carriage of inhalers for asthma treatment in secondary schools in North East England
Source: PeerJ. 2016 May 5;4:e2006. doi: 10.7717/peerj.2006 (PMC4860314; doi:10.7717/peerj.2006)
Supplement: File S1 — Questionnaire sent to school head teachers [file peerj-04-2006-s002.docx]

Asthma in schools survey

School:

Dear Head Teacher,

We would be very grateful if you could answer the following two questions and return this sheet in the stamped addressed envelope provided by 31st May 2015. Alternatively, you can complete this online at **www.asthmasurvey.net**

**1. What approach does your school take to inhalers for asthma?**

[ ] Pupils are automatically allowed to carry their own inhalers at all times

[ ] Pupils are allowed to carry their own inhalers after discussion and agreement with

the school on an individual basis

[ ] Inhalers are held by classroom staff (e.g. teachers)

[ ] Inhalers are stored in a single central location (e.g. with school nurse)

[ ] Another arrangement, or different arrangements according to pupils’ ages
(please describe in the box below)

|  |
| --- |

**2. Does your school have an emergency Salbutamol inhaler (blue / reliever inhaler) for use by any pupil in an asthma emergency?**

[ ] Yes

[ ] No

Many thanks for your time and help.
